# Editorial: Multidisciplinary perspectives on team sports: contextualizing training and competition demands

**DOI:** 10.3389/fpsyg.2024.1505156

**Published:** 2024-11-25

**Authors:** Pierpaolo Sansone, Vincenzo Rago, Miguel-Angel Gomez-Ruano

**Affiliations:** ^1^Department of Wellbeing, Nutrition and Sport, Pegaso Telematic University, Naples, Italy; ^2^Research Center for High Performance Sport, Catholic University of Murcia, Murcia, Spain; ^3^Faculty of Health and Sports Sciences, Universidad Europeia, Lisbon, Portugal; ^4^Facultad de Ciencias de la Actividad Física y el Deporte, Universidad Politécnica de Madrid, Madrid, Spain

**Keywords:** complex system (CS), situational variables, physical demands, athletic performance, physiological responses, technical-tactical analysis, perceptual measures

## Introduction

Team sports training and match-play require athletes to bring a mix of abilities to the table. They need to be physically strong and conditioned, tactically aware, technically skilled, mentally tough and socially intelligent. These abilities work together in a complex, interconnected way, affecting each other. Team sports research focuses on understanding what is needed in a game so that coaches and other performance support staff can assist athletes in developing the right skills and capacities. Given how complex team sports are, it's important to consider various factors from different areas—physical, tactical, and psychological among others—when analyzing performance.

## Game-based conditioning

One of the most stimulating and useful training methods in team sport is game-based conditioning, because it places athletes in scenarios that closely resemble competition from multitude perspectives (i.e. physical, technical-tactical, psychological and cognitive). It is therefore not surprising that three articles published in this topic focus on game-based conditioning, and specifically small-sided games (SSGs). As found by Zeng et al. basketball SSGs elicited similar physiological (heart rate) and perceptual responses (rating of perceived exertion) than high-intensity interval training (HIIT) across a 4-week intervention while it was enjoyed substantially more than HIIT by female athletes. These findings indicate that basketball practitioners can be confident in incorporating SSGs to players' adherence and motivation in training. Additionally, Palao et al. manipulated the rules related to net height, serve and court size in under-14 female volleyball tournaments, observing differences in technical-tactical variables (i.e. serves, attacks, blocks) of the game development according to specific constraints, while no differences were found for the physical demands (i.e. external load). Furthermore, Ueda et al. conducted a systematic review about how the number of players affects creativity in soccer SSGs training. The authors found that reducing the number of players and the court size facilitates exploratory behavior, variability and creative actions, which should be considered by soccer practitioners to implement these essential players' abilities.

## Psychological aspects in team sports

Five studies addressed psychological aspects of team sport performance. A meta-analysis by Kwon quantified the effectiveness of team building interventions on team cohesion and found greater effectiveness for participants aged 15–20 years old competing at collegiate level, and engaged in interventions longer than 2 weeks. An interesting paper by Aizava et al. demonstrated significant relationships between self-efficacy and mental toughness with sport-specific performance outcomes such as wins, red cards and wrong passes. These novel findings on the relationship between psychological characteristics and technical-tactical aspects further strengthen the need for multidisciplinary approaches as suggested by this Research Topic. The study by Aprò et al. validated a questionnaire on grit traits in a sample of Hungarian athletes and found that national team athletes had higher grit scores, suggesting that this personality trait should be considered for talent identification and athlete selection. Reinke and Schmitz found that both children and pre-adolescents were able to rate their perceived effort during soccer training, with better abilities in 13-years old than 11-years old players due to their higher cognitive abilities thanks to their higher cognitive development, which suggests the relationships between cognitive and perceptual aspects of team sport performance. In padel players, Conde-Ripoll et al. found heightened self-confidence before competitive matches than training for both higher and lower-level players, while somatic anxiety was higher before competition than training only for higher-level players. The authors suggest that self-confidence and somatic anxiety should be monitored in padel practitioners, and a sport psychologist might help improve the players' mental skills to optimize their preparedness for the mentally demanding competitive matches.

## Contextual and positional factors influencing basketball game performance

Two studies evaluated team and players' performances in the Chinese Basketball Association (CBA). Qiu et al. evaluated momentum, an important constraint that can significantly impact the game outcome in basketball. The study found higher occurrences of momentum for winning teams compared to losing ones; additionally, the game quarter is an important game part on which teams should perform well, especially for weaker team facing stronger teams. Chen et al. focused on the individual player and their impact on team offensive performances. The study described 14 offensive roles for native players and five for foreign players, with the performances of spot-up wings who attack and bigs who cut to the rim which significantly influence team's performance. These two basketball studies provide valuable information for coaches on how to design the team's tactical strategy by manipulating efforts across the game duration as well as players' roles.

## Conclusions

Altogether, the studies published in this Research Topic have successfully covered relevant multidimensional aspects of team sport performance. These findings related to game-based conditioning, psychological traits and game-related contextual and positional factors have been demonstrated to significantly impact players' and team performances and should therefore be well considered by team sport coaches as they can increase the team's chances of success. Specifically, (1) SSGs can be implemented to increase players' enjoyment, creativity, and to manipulate their physical demands; (2) team cohesion, self-efficacy, self-confidence, mental toughness and anxiety should be well considered by team sport coaches as these psychological traits are associated with success in competition; and (3) in the CBA basketball league, generating momentum and improving performances of wings and centers can increase the chances of success. Overall, the studies in this Research Topic have explored several important factors that influence how teams and players perform. Coaches should take these insights into account to improve training, helping their teams perform better and increase their chances of winning.

